# A Green Ultrasound Synthesis, Characterization and Antibacterial Evaluation of 1,4-Disubstituted 1,2,3-Triazoles Tethering Bioactive Benzothiazole Nucleus

**DOI:** 10.3390/molecules21040505

**Published:** 2016-04-18

**Authors:** Nadjet Rezki

**Affiliations:** 1 Department of Chemistry, Faculty of Sciences, Taibah University, Al-Madinah Al-Munawarah 30002, Saudi Arabia; nadjetrezki@yahoo.fr; Tel.: +966-540-953-573; 2 Laboratoire de Chimie & Electrochimie des Complexes Métalliques (LCECM), USTO-MB, Department of Chemistry, Faculty of Sciences, University of Sciences, and Technology Mohamed Boudiaf, B.p. 1505 El M′nouar, Oran 31000, Algeria

**Keywords:** 1,2,3-triazole, benzothiazole, 1,3-dipolar cycloaddition, ultrasound, antimicrobial activity

## Abstract

The synthesis of *N*-(benzo[*d*]thiazol-2-yl)-2-(4-substituted-1*H*-1,2,3-triazol-1-yl)acetamides **5a**–**r** via the 1,3-dipolar cycloaddition reaction between 2-azido-*N*-(benzo[*d*]thiazol-2-yl)acetamide derivatives **3a**–**c** and different alkynes were performed in the presence and absence of ultrasound irradiation. The synthesis was carried out using *t*-BuOH/H_2_O (1:1, *v*/*v*) as reaction solvents and CuSO_4_·5H_2_O/sodium ascorbate as the catalyst. The copper catalyst was implemented to provide the regioselective 1,4-disubstituted 1,2,3-triazoles **5a**–**r**. Significant reductions in reaction times with comparably higher yields were observed when the reactions were carried out under ultrasound irradiation. The structures of the newly synthesized 1,2,3-triazoles were elucidated by IR, NMR, MS, and elemental analyses. They were also screened for their antimicrobial activity against three gram-positive (*Streptococcus pneumonia*, *Bacillus subtilis*, and *Staphylococcus aureus*), three gram-negative (*Pseudomonas* *aeuroginosa*, *Escherichia coli*, and *Klebsiella pneumonia*), and two fungal strains (*Aspergillus fumigates* and *Candida albicans*). Most of the tested compounds displayed promising antimicrobial activities at a Minimum Inhibition Concentration (MIC) of 4–16 μg/mL.

## 1. Introduction

Since the conception of “click chemistry” by Sharpless and co-workers, the synthesis of 1,2,3-triazoles is well-known and has been thoroughly studied [1]. In these reactions, the 1,3-dipolar cycloaddition between organoazides and terminal alkynes carried out under copper-catalyzed conditions have been reported to afford the regioisomeric 1,4-disubstituted 1,2,3-triazoles as the single regioismers [2].

The 1,2,3-triazole core has been recognized as one of the most potent azoles with broad chemotherapeutic properties including antifungal [3], anticancer [4], antitubercular [5], antimalarial [6], anti-inflammatory [7], and antiviral [8] activities, along with the application of the concept of “click-synthesis” for their efficient and quick synthesis.

Benzothiazole and its derivatives have been widely recognized as privileged scaffolds in drug design associated with a wide spectrum of medicinal applications [9,10,11]. These include antimicrobial [12,13,14,15,16], anticancer [17,18,19,20], anthelmintic [21], and antidiabetic [22] activities.

Owing to the interesting advantages of ultrasound in modern heterocyclic synthesis, this eco-friendly approach has been the focus of numerous investigations by several research groups for the design of novel potentially active heterocycles [23,24]. The ultrasound method can serve not only as a good alternative but also facilitate reaction in shorter times with higher yields [25,26].

In order to evaluate the synergistic effect of these heterocyclic moieties in a single molecular framework, and in continuation of the author’s effort to design structurally diverse bioactive polyheterocyclic systems [27,28,29], reported herein is an eco-friendly click synthesis of a library of new regioselective 1,4-disubstituted 1,2,3-triazoles linked to a bioactive benzothiazole moiety through an acetamide linkage under both ultrasound and conventional thermal heating based on literature data [30,31,32,33,34]. The newly synthesized compounds were also subjected to an *in vitro* antimicrobial screening against several clinical bacterial and fungal strains. 

## 2. Results and Discussion

### 2.1. Chemistry

**Scheme 1:**
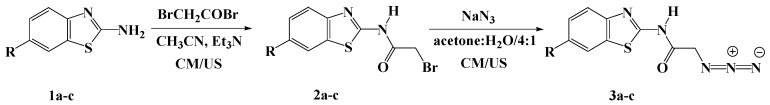
Synthesis of azidobenzothiazoles **3a**–**c** under ultrasound (US) and conventional methods (CM).

**Scheme 2:**
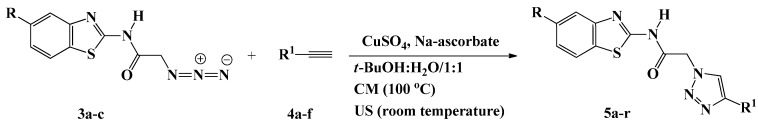
Synthesis of 1,2,3-triazoles **5a**–**r** based benzothiazole.

The desired 1,2,3-triazoles-based benzothiazoles were designed and synthesized starting from 2-aminobenzothiazole derivatives **1a**–**c** as outlined in Scheme 1 and Scheme 2.

Compounds **2a**–**c** were employed for the synthesis of the starting azidobenzothiazoles **3a**–**c**, needed for the 1,3-dipolar cycloaddition reaction. The synthesis of *N*-(benzo[*d*]thiazol-2-yl)-2-bromoacetamides **2a**–**c** was carried out based on a reported procedure [35] with slight modifications, as depicted in Scheme 1. Thus, compounds **2a**–**c** were synthesized in 83%–88% yields via the acylation of un/substituted aminobenzothiazole **1a**–**c** with bromoacetyl bromide, in the presence of triethylamine in acetonitrile at room temperature. The ultrasound irradiation was also used to construct the same products **2a**–**c** in shorter time (1 h) with higher product yields (89%–92%) compared to the conventional methods (Table 1).

The treatment of **2a**–**c** with sodium azide in a mixture of acetone:water (4:1, *v*/*v*) at room temperature for 24 h afforded the corresponding azidobenzothiazoles **3a**–**c** in good to excellent yields (88%–92%), as shown in Scheme 1. It should be noted that compound **3a** has been previously synthesized in 90% yield from the alkylation of **1a** with the appropriate acid chloride in the presence of triethylamine in dimethylformamide (DMF), followed by nucleophilic substitution with sodium azide [36].

Alternatively, the ultrasound irradiation greatly accelerated the reaction rates under the same reaction conditions. Only 2–3 h were needed to furnish the azido compounds **3a**–**c** in high yields (Table 1).

Initial formation of the *N-*(benzo[*d*]thiazol-2-yl)-2-bromoacetamides **2a**–**c** was unambiguously confirmed by their spectroscopic data. Their IR spectra clearly showed the disappearance of the amino group and the appearance of strong absorptions near 1695–1700 cm^−1^ and 3277–3325 cm^−1^ characteristic of the carbonyl (C=O) and the amide (NH) group, respectively. Moreover, the ^1^H-NMR spectra exhibited characteristic singlets at δ_H_ 4.20–4.27 ppm due to the methylene protons. The amide protons (NH) were observed at δ_H_ 6.42–6.48 ppm. The aromatic protons resonated at their expected chemical shift of δ_H_ 7.22–8.64 ppm. In addition, the ^13^C-NMR spectra revealed characteristic signals between δ_C_ 28.5–41.7 ppm belonging to the methylene groups. The spectra also revealed the appearance of new signals at 165.4–166.7 ppm attributed to the carbonyl groups, which is another piece of evidence of the incorporation of the acetyl moiety in their structures.

The structures of the synthesized azido derivatives **3a**–**c** were also illustrated on the basis of their spectroscopic data. Their IR spectra exhibited an absorption band near 2105–2122 cm^−1^, confirming the presence of the azido group in their structures. Compounds **3a**–**c** exhibited practically similar ^1^H-NMR and ^13^C-NMR spectral patterns to those observed for their precursors **2a**–**c** (See experimental section).

Huisgen copper(I)-catalyzed 1,3-dipolar cycloadditions between the azido benzothiazoles **3a**–**c** and the appropriate terminal alkynes **4a**–**f** were performed under both conventional and ultrasound conditions to generate **5a**–**r**, a new library of 1,4-disubstiuted 1,2,3-triazoles linked via an acetamide connecting unit to benzothiazoles (Scheme 2). The easy access to a variety of acetylenic compounds (**4a**–**f**) allowed the incorporation of several functionalities into the triazole scaffold of **5a**–**r**. 

The reaction required heating at 100 °C for 6–10 h, in the presence of a catalytic amount of copper sulfate (CuSO_4_) and sodium ascorbate using *t*-BuOH/H_2_O (1:1, *v*/*v*) as solvents. Whereas only 3–6 h were needed to give the same products in comparable yields when the reactions were assisted by ultrasound irradiation at room temperature (Table 2). In the present study, CuSO_4_/sodium ascorbate was selected as the catalyst system to yield the regioisomeric 1,4-disubstituted 1,2,3-triazoles as the sole regioisomer product. These results were in agreement with those previously reported for the successful green ultrasound-assisted synthesis of 1,4-disubstituted 1,2,3-triazoles, where the best results were obtained when using *t*-BuOH/H_2_O as the medium and CuSO_4_/sodium ascorbate as the catalyst [30,31,32,33,34].

All of the newly synthesized triazoles **5a**–**r** were fully characterized using IR, NMR, and MS and elemental analysis. Their structures were in accordance with their spectroscopic properties. Their IR spectra showed the disappearance of the absorption bands attributed to the azido and the acetylenic groups of the corresponding starting materials, which confirmed their involvement in the cycloaddition reaction. 

Moreover, the ^1^H-NMR spectra showed the absence of the acetylenic protons and the appearance of the diagnostic singlets at δ_H_ 7.72–8.71 and δ_H_ 12.67–12.99 ppm attributed to the triazolic proton and the amidic NH proton, respectively, supporting the proposed 1,2,3-triazole structures. In addition, their ^13^C-NMR spectra were consistent with the designed structures, the disappearance of the sp carbons provided additional evidence for the success of the cycloaddition reaction. In addition, the ^1^H-NMR spectra of compounds **5a**–**c**, **5g**–**i**, and **5m**–**o** revealed the presence of five and/or ten extra aromatic protons, depending on the nature of substitution on N-1 of the triazole ring. The characteristic signals at δ_H_ 4.50–6.80 ppm were attributed to the 1,2,3-triazole derivatives carrying the hydroxy group. 

In addition, all aliphatic protons and carbons resonated at the expected chemical shifts in the ^1^H-NMR spectra of triazoles **5d**, **5j** and **5p**, the trimethylsilyl group appeared at δ_H_ 0.28–0.34 ppm corresponding to nine protons, and at δ_C_ 0.3–0.5 ppm in the ^13^C-NMR spectra for the three methyl groups.

### 2.2. Biology

#### Antimicrobial Activity

The antibacterial and antifungal inhibition effects of the title compounds **2a**–**c**, **3a**–**c** and **5a**–**r** against a panel of pathogenic bacterial and fungal strains were determined using the broth dilution method [37,38]. The Minimum Inhibition Concentration (MIC) screening results are given in Table 3, and Ciprofloxacin and Fluconazole were used as control drugs. The antimicrobial screening results revealed that the precursors **2a**–**c** used for further derivatization were basically inactive against all of the fungal strains, but exhibited modest antibacterial activity at MIC 16–31.25 μg/mL.

The azidolysis of compounds **2a**–**c** to their corresponding azido benzothiazoles **3a**–**c** caused no enhancement of the antibacterial and antifungal activities. However, as expected, the 1,2,3-triazoles **5a**–**r** were more potent than the corresponding azidobenzothiazoles **3a**–**c**. Among them, *N*-1 hydroxylated alkyl substituted triazoles **5e**, **5f**, **5k**, **5l**, **5q,** and **5r** exhibited the highest antibacterial activity at MIC 4–8 μg/mL and antifungal activity at MIC 4–16 μg/mL. 

Moreover, the presence of a sulfonyl group in the benzothiazole residue of compounds **4a**–**r** was found to significantly increase the antimicrobial activity towards all of the tested pathogenic strains at MIC 4–16 μg/mL.

Based on the preliminary structure-activity relationship analysis, it can be stated that the incorporation of a 1,2,3-triazole nucleus in the benzothiazole structure significantly improved the antimicrobial activities of the resulting biheterocyclic systems **5a**–**r**, especially when the 1,2,3-triazole was substituted with hydroxylated alkyl side chain at position 1. In addition, it must be noted that among all of the tested compounds, those with methylsulfonyl substitution in the benzothiazole ring **5q** and **5r** exhibited the highest inhibition against all of the tested microorganisms.

## 3. Experimental Section

### 3.1. General

All melting points were measured on a variable heater (Stuart, UK) melt-temp apparatus and are uncorrected. Sonochemical reactions were performed in a Kunshan KQ-250B ultrasound cleaner (50 KHz, 240 W, Kunshan, China). The NMR spectra were measured with an Avance Bruker spectrotometer (Fällanden, Switzerland) at 400 MHz for the ^1^H-NMR analysis and at 100 MHz for the ^1^H-NMR analysis, using Tetramethylsilane (TMS) (0.00 ppm) as the internal standard and DMSO-*d*_6_ as a solvent. The IR spectra were measured in a KBr matrix with a Perkin-Elmer 1430 series FTIR spectrometer (Boston, MA, USA). A Finnigan MAT 95XL spectrometer (Darmstadt, Germany) was used for the determination of the EI mass spectra. Elemental analyses were performed using a GmbH-Vario EL III Element Analyzer (Munich, Germany). 

### 3.2. General Procedure for the Synthesis of 2-Bromo-N-(6-un/substitutedbenzo[d]thiazol-2-yl)acetamide ***2a**–**c***

**Method a:** Bromoacetyl bromide (1.2 mmol) was added dropwise to a mixture of 2-aminobenzothiazole derivatives **1a**–**c** (1 mmol) and triethylamine (1.2 mmol) in acetonitrile (15 mL) at room temperature with stirring. The stirring was continued at room temperature for 2–3 h. The *N*-(benzo[*d*]thiazol-2-yl)-2-bromoacetamide thus formed was filtered, washed with water, and recrystallized from ethanol.

**Method b:** A mixture of 2-aminobenzothiazole derivatives **1a**–**c** (1 mmol) in acetonitrile (15 mL), triethylamine (1.2 mmol) and bromoacetyl bromide (1.2 mmol) was sonicated for 1 h at room temperature under inert atmosphere in a laboratory ultrasonic cleaning bath. The reaction was treated as described above. 

*N-(Benzo[d]thiazol-2-yl)-2-bromoacetamide* (**2a**). Colorless needles, m.p. 175–176 °C; (Lit. m.p. 1730–174 °C) [35]. IR (*υ*, cm^−1^): 1580 (C=C), 1625 (C=N), 1695 (C=O), 2948 (C-H al), 3053 (C-H ar), 3290 (N-H). ^1^H-NMR: δ 4.26 (s, 2H, C**H**_2_), 6.43 (bs, 1H, N**H**), 7.30–7.36 (m, 1H, Ar-**H**), 7.43–7.49 (m, 1H, Ar-**H**), 7.75–7.80 (m, 1H, Ar-**H**), 7.99–8.03 (m, 1H, Ar-**H**). ^13^C-NMR: δ 28.5 (**C**H_2_), 120.6, 121.7, 123.7, 126.2, 131.4, 148.3, 157.5, 166.0 (Ar-**C**, **C**=N, C=O) ppm. EI MS (*m*/*z*): 269.81 (M^+^). Anal. Calcd for C_9_H_7_BrN_2_OS: C 39.87; H 2.60; N 10.33. Found: C 39.72; H 2.68; N 10.40.

*2-Bromo-N-(6-methylbenzo[d]thiazol-2-yl)acetamide* (**2b**). Colorless needles, m.p. 157–158 °C. IR (*υ*, cm^−1^): 1564 (C=C), 1610 (C=N), 1700 (C=O), 2925 (C-H al), 3084 (C-H ar), 3277 (N-H). ^1^H-NMR: δ 2.44 (s, 3H, C**H**_3_), 4.20 (s, 2H, C**H**_2_), 6.48 (s, 1H, N**H**), 7.22 (d, 1H, *J* = 8 Hz, Ar-**H**), 7.63 (d, 1H, *J* = 8 Hz, Ar-**H**), 7.75 (s, 1H, Ar-**H**). ^13^C-NMR: δ 20.8 (**C**H_3_), 31.6 (**C**H_2_), 120.4, 122.6, 123.2, 126.0, 127.8, 131.4, 147.4, 165.4 (Ar-**C**, **C**=N, **C**=O) ppm. EI MS (*m*/*z*): 284.06 (M^+^). Anal. Calcd for C_10_H_9_BrN_2_OS: C 42.12; H, 3.18; N, 9.82. Found: C 42.12; H, 3.18; N, 9.82.

*2-Bromo-N-(6-(methylsulfonyl)benzo[d]thiazol-2-yl)acetamide* (**2c**). Colorless needles, m.p. 211–212 °C. IR (υ, cm^−1^): 1572 (C=C), 1628 (C=N), 1698 (C=O), 2970 (C-H al), 3019 (C-H ar), 3325 (N-H). ^1^H-NMR: δ 3.20 (s, 3H, C**H**_3_), 4.27 (s, 2H, C**H**_2_), 6.42 (bs, 1H, N**H**), 7.84 (d, 1H, *J* = 8 Hz, Ar-**H**), 7.94 (d, 1H, *J* = 8 Hz, Ar-**H**), 8.64 (s, 1H, Ar-**H**). ^13^C-NMR: δ 32.5 (**C**H_3_), 41.7 (**C**H_2_), 122.5, 123.8, 126.0, 128.1, 129.9, 144.6, 152.8, 166.7 (Ar-**C**, **C**=N, **C**=O) ppm. EI MS (*m*/*z*): 347.79 (M^+^). Anal. Calcd for C_10_H_9_BrN_2_O_3_S_2_: C 34.39; H 2.60; N 8.02. Found: C 34.30; H 2.48; N 8.15.

### 3.3. General Procedure for the Synthesis of Azido Benzothiazole Derivatives ***3a**–**c***

**Method a:** A mixture of compounds **2a**–**c** (1 mmol) and sodium azide (1.2 mmol) in a mixture of acetone:water (4:1) (10 mL) was stirred for 24 h at room temperature. The excess of solvent was evaporated under vacuum. Products **3a**–**c** were collected by filtration, washed with water and recrystallized from ethanol. 

**Method b:** A mixture of compounds **2a**–**c** (1 mmol) and sodium azide (1.2 mmol) in a mixture of acetone:water (4:1) (10 mL) was sonicated for 2–3 h at room temperature under inert atmosphere in a laboratory ultrasonic cleaning bath. The reaction mixture was treated as described above. 

*2-Azido-N-(benzo[d]thiazol-2-yl)acetamide* (**3a**). Colorless needles, m.p. 210–211 °C; (Lit. m.p. 208–210 °C [36]). IR (*υ*, cm^−1^): 1572 (C=C), 1617 (C=N), 1698 (C=O), 2105 (-N=N=N), 2923 (C-H al), 3076 (C-H ar), 3315 (N-H). ^1^H-NMR: δ 4.26 (s, 2H, C**H**_2_), 7.36 (dd, 1H, *J* = 4, 12 Hz, Ar-**H**), 7.47 (dd, 1H, *J* = 4, 12 Hz, Ar-**H**), 7.78 (d, 1H, *J* = 8 Hz, Ar-**H**), 8.01 (d, 1H, *J* = 8 Hz, Ar-**H**), 12.57 (s, 1H, N**H**). ^13^C-NMR: δ 49.2 (**C**H_2_), 119.1, 120.2, 122.2, 124.6, 129.9, 147.2, 155.9, 166.1 (Ar-**C**, **C**=N, **C**=O) ppm. EI MS (*m*/*z*): 233.15 (M^+^). Anal. Calcd for C_9_H_7_N_5_OS: C 46.34; H 3.02; N 30.03. Found: C 46.51; H 3.14; N 30.20.

*2-Azido-N-(6-methylbenzo[d]thiazol-2-yl)acetamide* (**3b**). Colorless needles, m.p. 229–230 °C. IR (υ, cm^-1^): 1572 (C=C), 1603 (C=N), 1701 (C=O), 2122 (-N=N=N), 2955 (C-H al), 3027 (C-H ar), 3294 (N-H). ^1^H-NMR: δ 2.42 (s, 3H, C**H**_3_), 4.24 (s, 2H, C**H**_2_), 7.20 (d, 1H, *J* = 8 Hz, Ar-**H**), 7.67 (d, 1H, *J* = 8 Hz, Ar-**H**), 7.71 (s, 1H, Ar-**H**), 12.75 (s, 1H, N**H**). ^13^C-NMR: δ 21.1 (**C**H_3_), 33.2 (**C**H_2_), 120.8, 122.4, 123.6, 127.4, 127.9, 131.2, 148.8, 165.8 (Ar-**C**, **C**=N, **C**=O) ppm. EI MS (*m*/*z*): 246.97 (M^+^). Anal. Calcd for C_10_H_9_N_5_OS: C 48.57; H 3.67; N 28.32. Found: C 48.44; H 3.58; N 28.43.

*2-Azido-N-(6-(methylsulfonyl)benzo[d]thiazol-2-yl)acetamide* (**3c**). Colorless needles, m.p. 242–243 °C. IR (υ, cm^−1^): 1585 (C=C), 1616 (C=N), 1713 (C=O), 2105 (-N=N=N), 2939 (C-H al), 3064 (C-H ar), 3307 (N-H). ^1^H-NMR: δ 3.16 (s, 3H, C**H**_3_), 4.32 (s, 2H, C**H**_2_), 7.86–7.96 (m, 2H, Ar-**H**), 8.60 (s, 1H, Ar-**H**), 12.81 (s, 1H, N**H**). ^13^C-NMR: δ 38.7 (**C**H_3_), 50.9 (**C**H_2_), 122.4, 124.2, 129.4, 131.7, 134.4, 145.8, 153.3, 166.0 (Ar-**C**, **C**=N, **C**=O) ppm. EI MS (*m*/*z*): 311.18 (M^+^). Anal. Calcd for C_10_H_9_N_5_O_3_S_2_: C 38.58; H 2.91; N 22.49. Found: C 38.69; H 2.98; N 22.62.

### 3.4. General Procedures for the Click Synthesis of 1,2,3-Triazoles ***5a**–**r***

**Method a:** To a stirring solution of equimolar amounts of azidobenzothiazole **3a**–**c** and terminal alkyne **4a–f** dissolved in *t*-BuOH and water (1:1), CuSO_4_ (0.01 eq) and Na-ascorbate (0.01 eq) were added. Stirring was continued for 6–10 h at 100 °C, until the consumption of the starting material as indicated by thin layer chromatography (TLC). Saturated brine solution was added to the reaction mixture, then the crude was extracted with ethyl acetate (3 × 50 mL) and dried over sodium sulfate. Removal of the solvent in vacuum gave the desired 1,2,3-triazole derivatives **5a**–**r** which were crystallized from ethanol. 

**Method b:** A mixture of equimolar amounts of azidobenzothiazole **3a**–**c** and terminal alkyne **4a**–**f**, CuSO_4_ (0.01 eq) and Na-ascorbate (0.01 eq) in *t*-BuOH and water (1:1) was sonicated for 3–6 h at room temperature under inert atmosphere in a laboratory ultrasonic cleaning bath. The reaction mixture was treated as described above.

*N-(Benzo[d]thiazol-2-yl)-2-(4-phenyl-1H-1,2,3-triazol-1-yl)acetamide* (**5a**). Brown solid, m.p. 122–123 °C. IR (υ, cm^−1^): 1566 (C=C), 1607 (C=N), 1695 (C=O), 2965 (C-H al), 3038 (C-H ar), 3304 (N-H). ^1^H-NMR: δ 5.61 (s, 2H, C**H**_2_), 7.31–7.38 (m, 2H, Ar-**H**), 7.44–7.49 (m, 3H, Ar-**H**), 7.81 (d, 1H, *J* = 8 Hz, Ar-**H**), 7.90 (d, 2H, *J* = 8 Hz, Ar-**H**), 8.01 (d, 1H, *J* = 8 Hz, Ar-**H**), 8.65 (s, 1H, C**H**-1,2,3-triazole), 12.99 (s, 1H, N**H**). ^13^C-NMR: δ 51.7 (**C**H_2_), 120.6, 121.8, 123.1, 123.8, 125.1, 126.2, 127.9, 128.9, 130.5, 131.5, 146.3, 157.8, 165.8 (Ar-**C**, **C**=N, **C**=O) ppm. EI MS (*m*/*z*): 335.18 (M^+^). Anal. Calcd for C_17_H_13_N_5_OS: C 60.88; H 3.91; N 20.88. Found: C 60.69; H 3.86; N 20.76.

*N-(Benzo[d]thiazol-2-yl)-2-(4-(hydroxydiphenylmethyl)-1H-1,2,3-triazol-1-yl)-acetamide* (**5b**). Brown solid, m.p. 146–147 °C. IR (υ, cm^−1^): 1583 (C=C), 1615 (C=N), 1706 (C=O), 2942 (C-H al), 3079 (C-H ar), 3314–3389 (N-H, O-H). ^1^H-NMR: δ 5.53 (s, 2H, C**H**_2_), 6.59 (s, 1H, O**H**), 7.00–7.38 (m, 12H, Ar-**H**), 7.79–8.00 (m, 3H, C**H**-1,2,3-triazole, Ar-**H**), 12.82 (s, 1H, N**H**). ^13^C-NMR: δ 49.4 (**C**H_2_), 64.7 (**C**-OH), 119.3, 120.6, 121.2, 121.6, 122.7, 123.0, 123.9, 124.4, 125.3, 126.4, 128.2, 128.4, 131.7, 133.2, 147.1, 156.5, 166.3 (Ar-**C**, **C**=N, **C**=O) ppm. EI MS (*m*/*z*): 441.02 (M^+^). Anal. Calcd for C_24_H_19_N_5_O_2_S: C 65.29; H 4.34; N 15.86. Found: C 65.11; H 4.22; N 15.79.

*N-(Benzo[d]thiazol-2-yl)-2-(4-(hydroxy(phenyl)methyl)-1H-1,2,3-triazol-1-yl)-acetamide* (**5c**). Brown solid, m.p. 180–181 °C. IR (υ, cm^−1^): 1573 (C=C), 1602 (C=N), 1711 (C=O), 2924 (C-H al), 3082 (C-H ar), 3287–3355 (N-H, O-H). ^1^H-NMR: δ 5.42 (s, 2H, C**H**_2_), 5.81 (d, 1H, *J* = 8 Hz, C**H**), 5.97 (d, 1H, *J* = 8 Hz, O**H**), 7.19–7.36 (m, 7H, Ar-**H**), 7.72–7.92 (m, 3H, C**H**-1,2,3-triazole, Ar-**H**), 12.75 (s, 1H, N**H**). ^13^C-NMR: δ 49.4 (**C**H_2_), 64.7 (**C**-OH), 119.6, 120.8, 121.1, 122.8, 123.2, 124.7, 125.9, 126.7, 128.8, 131.5, 132.0, 146.6, 156.8, 166.5 (Ar-**C**, **C**=N, **C**=O) ppm. EI MS (*m*/*z*): 365.22 (M^+^). Anal. Calcd for C_18_H_15_N_5_O_2_S: C 59.16; H 4.14; N 19.17. Found:C 59.45; H 4.26; N 19.43.

*N-(Benzo[d]thiazol-2-yl)-2-(4-(trimethylsilyl)-1H-1,2,3-triazol-1-yl)acetamide* (**5d**). Brown solid, m.p. 108–109 °C. IR (υ, cm^−1^): 1580 (C=C), 1602 (C=N), 1701 (C=O), 2965 (C-H al), 3051 (C-H ar), 3313 (N-H). ^1^H-NMR: δ 0.29 (s, 9H, 3 × C**H**_3_), 5.56 (s, 2H, C**H**_2_), 7.29–7.33 (m, 1H, Ar-**H**), 7.40–7.46 (m, 1H, Ar-**H**), 7.81 (d, 1H, *J* = 8 Hz, Ar-**H**), 7.99 (d, 1H, *J* = 8 Hz, Ar-**H**), 8.20 (s, 1H, C**H**-1,2,3-triazole), 12.89 (s, 1H, NH). ^13^C-NMR: δ 0.5 (**C**H_3_), 50.9 (**C**H_2_), 120.3, 121.6, 122.8, 123.9, 128.4, 129.8, 147.5, 155.3, 166.7 (Ar-**C**, **C**=N, **C**=O) ppm. EI MS (*m*/*z*): 331.24 (M^+^). Anal. Calcd for C_14_H_17_N_5_OSSi: C 50.73; H 5.17; N 21.13. Found: C 50.41; H 5.05; N 21.33.

*N-(Benzo[d]thiazol-2-yl)-2-(4-(2-hydroxyethyl)-1H-1,2,3-triazol-1-yl)acetamide* (**5e**). Brown solid, m.p. 228–229 °C. IR (υ, cm^−1^): 1574 (C=C), 1615 (C=N), 1706 (C=O), 2938 (C-H al), 3060 (C-H ar), 3302–3389 (N-H, O-H). ^1^H-NMR: δ 2.81 (t, 2H, *J* = 8 Hz, C-C**H**_2_), 3.67 (t, 2H, *J* = 8 Hz, OC**H**_2_), 4.72 (s, 1H, O**H**), 5.50 (s, 2H, C**H**_2_), 7.32–7.46 (m, 2H, Ar-**H**), 7.80–7.95 (m, 3H, C**H**-1,2,3-triazole, Ar-H), 12.86 (s, 1H, N**H**). ^13^C-NMR: δ 29.0 (C-**C**H_2_), 50.4 (**C**H_2_), 60.3 (O**C**H_2_), 120.8, 121.3, 122.6, 123.1, 127.7, 129.3, 146.1, 156.7, 165.4 (Ar-**C**, **C**=N, **C**=O) ppm. EI MS (*m*/*z*): 303.15 (M^+^). Anal. Calcd for C_13_H_13_N_5_O_2_S: C 51.47; H 4.32; N 23.09. Found: C 51.62; H 4.28; N 23.18.

*N-(Benzo[d]thiazol-2-yl)-2-(4-(3-hydroxypropyl)-1H-1,2,3-triazol-1-yl)acetamide* (**5f**). Brown solid, m.p. 240–241 °C. IR (υ, cm^−1^): 1566 (C=C), 1611 (C=N), 1697 (C=O), 2918 (C-H al), 3045 (C-H ar), 3274–3364 (N-H, O-H). ^1^H-NMR: δ 1.76-1.79 (m, 2H, OCH_2_C**H**_2_), 2.69 (t, 2H, *J* = 8 Hz, C-C**H**_2_), 3.47 (b, 2H, OC**H**_2_ overlapped with DMSO), 4.50 (s, 1H, O**H**), 5.48 (s, 2H, C**H**_2_), 7.34 (dd, 1H, *J* = 4,8 Hz, Ar-**H**), 7.47 (dd, 1H, *J* = 4, 8 Hz, Ar-**H**), 7.79 (d, 1H, *J* = 8 Hz, Ar-**H**), 7.92 (s, 1H, C**H**-1,2,3-triazole), 7.99 (d, 1H, *J* = 4 Hz, Ar-**H**), 12.81 (s, 1H, N**H**). ^13^C-NMR: δ 29.0 (OCH_2_**C**H_2_), 32.1 (C-**C**H_2_), 51.4 (**C**H_2_), 60.0 (O**C**H_2_), 120.7, 121.7, 123.7, 123.9, 126.3, 129.4, 146.2, 156.0, 165.7 (Ar-**C**, **C**=N, **C**=O) ppm. EI MS (*m*/*z*): 317.21 (M^+^). Anal. Calcd for C_14_H_15_N_5_O_2_S: C 52.98; H 4.76; N 22.07. Found: C 52.80; H 4.59; N 22.28.

*N-(6-Methylbenzo[d]thiazol-2-yl)-2-(4-phenyl-1H-1,2,3-triazol-1-yl)acetamide* (**5g**). Brown solid, m.p. 133–134 °C. IR (υ, cm^−1^): 1560 (C=C), 1616 (C=N), 1703 (C=O), 2946 (C-H al), 3077 (C-H ar), 3345 (N-H). ^1^H-NMR: δ 2.41 (s, 3H, C**H**_3_), 5.59 (s, 2H, C**H**_2_), 7.27–7.37 (m, 2H, Ar-**H**), 7.45–7.49 (m, 2H, Ar-**H**), 7.68 (d, 1H, *J* = 8 Hz, Ar-**H**), 7.78 (s, 1H, Ar-**H**), 7.89 (d, 2H, *J* = 8 Hz, Ar-**H**), 8.62 (s, 1H, C**H**-1,2,3-triazole), 12.86 (s, 1H, N**H**). ^13^C-NMR: δ 20.9 (**C**H_3_), 39.9 (**C**H_2_), 121.3, 123.0, 125.1, 127.5, 127.9, 128.6, 130.2, 133.7, 147.4, 154.1, 165.4 (Ar-**C**, **C**=N, **C**=O) ppm. EI MS (*m*/*z*): 349.22 (M^+^). Anal. Calcd for C_18_H_15_N_5_OS: C 61.87; H 4.33; N 20.04. Found: C 61.80; H 4.27; N 19.98.

*2-(4-(Hydroxydiphenylmethyl)-1H-1,2,3-triazol-1-yl)-N-(6-methylbenzo[d]thiazol-2-yl)acetamide* (**5h**). Brown solid, m.p. 148–149 °C. IR (υ, cm^−1^): 1562 (C=C), 1604 (C=N), 1701 (C=O), 2929 (C-H al), 3036 (C-H ar), 3287–3374 (N-H, O-H). ^1^H-NMR: δ 2.41 (s, 3H, C**H**_3_), 5.52 (s, 2H, C**H**_2_), 6.60 (s, 1H, O**H**), 7.22–7.39 (m, 12H, Ar-**H**), 7.77 (s, 1H, Ar-**H**), 7.92 (s, 1H, C**H**-1,2,3-triazole), 12.75 (s, 1H, N**H**). ^13^C-NMR: δ 20.9 (**C**H_3_), 49.0 (**C**H_2_), 75.6 (**C**-OH), 121.3, 125.0, 125.5, 126.7, 126.9, 127.5, 127.9, 128.5, 129.5, 133.3, 147.0, 153.7, 164.7 (Ar-**C**, **C**=N, **C**=O) ppm. EI MS (*m*/*z*): 455.29 (M^+^). Anal. Calcd for C_25_H_21_N_5_O_2_S: C 65.92; H 4.65; N 15.37. Found: C 65.78; H 4.53; N 15.26.

*2-(4-(Hydroxy(phenyl)methyl)-1H-1,2,3-triazol-1-yl)-N-(6-methylbenzo[d]thiazol-2-yl)acetamide* (**5i**). Brown solid, m.p. 162–163 °C. IR (υ, cm^−1^): 1570 (C=C), 1607 (C=N), 1701 (C=O), 2924 (C-H al), 3032 (C-H ar), 3275–3345 (N-H, O-H). ^1^H-NMR: δ 2.45 (s, 3H, C**H**_3_), 5.52 (s, 2H, C**H**_2_), 5.91 (s, 1H, *J* = 4 Hz, C**H**), 6.08 (s, 1H, *J* = 8 Hz, O**H**), 7.30-7.50 (m, 6H, Ar-**H**), 7.72 (d, 1H, *J* = 8 Hz, Ar-**H**), 7.81 (s, 1H, Ar-**H**), 7.98 (s, 1H, C**H**-1,2,3-triazole), 12.75 (s, 1H, N**H**). ^13^C-NMR: δ 20.0 (**C**H_3_), 50,6 (**C**H_2_), 67.0 (**C**-OH), 119.3, 120.4, 122.8, 125.4, 126.1, 126.6, 127.1, 127.2, 130.6, 132.4, 143.1, 150.5, 164.8 (Ar-**C**, **C**=N, **C**=O) ppm. EI MS (*m*/*z*): 379.00 (M^+^). Anal. Calcd for C_19_H_17_N_5_O_2_S: C 60.14; H 4.52; N 18.46. Found: C 60.26; H 4.65; N 18.61.

*N-(6-Methylbenzo[d]thiazol-2-yl)-2-(4-(trimethylsilyl)-1H-1,2,3-triazol-1-yl)-acetamide* (**5j**). Brown solid, m.p. 111–112 °C. IR (υ, cm^−1^): 1583 (C=C), 1614 (C=N), 1694 (C=O), 2940 (C-H al), 3083 (C-H ar), 3316 (N-H). ^1^H-NMR: δ 0.34 (s, 9H, 3 × C**H**_3_), 2.40 (s, 3H, C**H**_3_), 5.50 (s, 2H, C**H**_2_), 7.26 (d, 1H, *J* = 8 Hz, Ar-**H**), 7.63 (d, 1H, *J* = 8 Hz, Ar-**H**), 7.70 (s, 1H, Ar-**H**), 7.99 (s, 1H, C**H**-1,2,3-triazole), 12.84 (s, 1H, N**H**). ^13^C-NMR: δ 0.4 (Si-**C**H_3_), 20.4 (**C**H_3_), 49.8 (**C**H_2_), 120.3, 121.7, 123.6, 126.9, 130.3, 132.0, 146.7, 154.0, 165.9 (Ar-**C**, **C**=N, **C**=O) ppm. EI MS (*m*/*z*): 345.26 (M^+^). Anal. Calcd for C_15_H_19_N_5_OSSi: C 52.15; H 5.54; N 20.27. Found: C 52.43; H 5.43; N 20.39.

*2-(4-(2-Hydroxyethyl)-1H-1,2,3-triazol-1-yl)-N-(6-methylbenzo[d]thiazol-2-yl)-acetamide* (**5k**). Brown solid, m.p. 239–240 °C. IR (υ, cm^−1^): 1592 (C=C), 1603 (C=N), 1711 (C=O), 2965 (C-H al), 3019 (C-H ar), 3280–3375 (N-H, O-H). ^1^H-NMR: δ 2.40 (s, 3H, C**H**_3_), 2.82 (t, 2H, *J* = 8 Hz, C-C**H**_2_), 3.68 (bt, 2H, OC**H**_2_), 4.72 (bs, 1H, OH), 5.47 (s, 2H, C**H**_2_), 7.28 (d, 1H, *J* = 8 Hz, Ar-**H**), 7.67 (d, 1H, *J* = 8 Hz, Ar-**H**), 7.77 (s, 1H, Ar-**H**), 7.94 (s, 1H, CH-1,2,3-triazole), 12.77 (s, 1H, N**H**). ^13^C-NMR: δ 20.9 (**C**H_3_), 29.0 (C-**C**H_2_), 51.4 (**C**H_2_), 60.3 (O**C**H_2_), 120.3, 121.3, 124.1, 127.5, 131.5, 133.3, 147.3, 155.1, 165.7 (Ar-**C**, **C**=N, **C**=O) ppm. EI MS (*m*/*z*): 317.22 (M^+^). Anal. Calcd for C_14_H_15_N_5_O_2_S: C 52.98; H 4.76; N 22.07. Found: C 52.77; H 4.68; N 22.31.

*2-(4-(3-Hydroxypropyl)-1H-1,2,3-triazol-1-yl)-N-(6-ethylbenzo[d]thiazol-2-yl)-acetamide* (**5l**). Brown solid, m.p. 252–253 °C. IR (υ, cm^−1^): 1573 (C=C), 1604 (C=N), 1705 (C=O), 2945 (C-H al), 3067 (C-H ar), 3280–3358 (N-H, O-H). ^1^H-NMR: δ 1.73–1.80 (m, 2H, OCH_2_C**H**_2_), 2.39 (s, 3H, C**H**_3_), 2.68 (t, 2H, *J* = 8 Hz, C-C**H**_2_), 3.45 (b, 2H, OC**H**_2_ overlapped with DMSO), 4.53 (s, 1H, O**H**), 5.43 (s, 2H, C**H**_2_), 7.24 (d, 1H, *J* = 8 Hz, Ar-**H**), 7.62 (d, 1H, *J* = 8 Hz, Ar-**H**), 7.72 (s, 1H, Ar-**H**), 7.90 (s, 1H, C**H**-1,2,3-triazole), 12.67 (bs, 1H, N**H**). ^13^C-NMR: δ 20.9 (**C**H_3_), 21.6 (OCH_2_**C**H_2_), 32.2 (C-**C**H_2_), 51.9 (**C**H_2_), 60.0 (O**C**H_2_), 117.3, 120.0, 123.4, 126.4, 127.2, 132.7, 146.6, 156.0, 165.7 (Ar-**C**, **C**=N, **C**=O) ppm. EI MS (*m*/*z*): 330.97 (M^+^). Anal. Calcd for C_15_H_17_N_5_O_2_S: C 54.36; H 5.17; N 21.13. Found: C 54.58; H 5.06; N 21.28.

*N-(6-(Methylsulfonyl)benzo[d]thiazol-2-yl)-2-(4-phenyl-1H-1,2,3-triazol-1-yl)-acetamide* (**5m**). Brown solid, m.p. 142–143 °C. IR (*υ*, cm^−1^): 1571 (C=C), 1610 (C=N), 1699 (C=O), 2957 (C-H al), 3026 (C-H ar), 3314 (N-H). ^1^H-NMR: δ 2.98 (s, 3H, C**H**_3_), 5.49 (s, 2H, C**H**_2_), 7.26–7.34 (m, 3H, Ar-**H**), 7.42–7.55 (m, 2H, Ar-**H**), 7.98 (d, 1H, *J* = 8 Hz, Ar-**H**), 8.05 (d, 1H, *J* = 8 Hz, Ar-**H**), 8.17 (s, 1H, Ar-**H**), 8.68 (s, 1H, C**H**-1,2,3-triazole), 12.76 (s, 1H, N**H**). ^13^C-NMR: δ 43.1 (**C**H_3_), 48.2 (**C**H_2_), 120.7, 122.9, 123.0, 124.5, 125.7, 126.2, 128.8, 130.4, 130.6, 145.6, 150.7, 154.3, 164.1 (Ar-**C**, **C**=N, **C**=O) ppm. EI MS (*m*/*z*): 413.14 (M^+^). Anal. Calcd for C_18_H_15_N_5_O_3_S_2_: C 52.29; H 3.66; N 16.94. Found: C 52.21; H 3.55; N 16.85.

*2-(4-(Hydroxydiphenylmethyl)-1H-1,2,3-triazol-1-yl)-N-(6-(methylsulfonyl)benzo[d]-thiazol-2-yl)acetamide* (**5n**). Brown solid, m.p. 154–155 °C. IR (υ, cm^−1^): 1578 (C=C), 1601 (C=N), 1712 (C=O), 2944 (C-H al), 3060 (C-H ar), 3265–3349 (N-H, O-H). ^1^H-NMR: δ 3.21 (s, 3H, C**H**_3_), 5.46 (s, 2H, C**H**_2_), 6.49 (s, 1H, O**H**), 7.26–7.31 (m, 10H, Ar-**H**), 8.07 (d, 1H, *J* = 8 Hz, Ar-**H**), 8.21 (d, 1H, *J* = 8 Hz, Ar-**H**), 8.25 (s, 1H, Ar-**H**), 8.87 (s, 1H, C**H**-1,2,3-triazole), 12.68 (s, 1H, N**H**). ^13^C-NMR: δ 40.3 (**C**H_3_), 49.7 (**C**H_2_), 79.1 (**C**-OH), 119.7, 120.2, 121.7, 124.3, 125.8, 128.0, 129.7, 130.7, 144.6, 149.8, 151.1, 155.3, 163.4 (Ar-**C**, **C**=N, **C**=O) ppm. EI MS (*m*/*z*): 519.02 (M^+^). Anal. Calcd for C_25_H_21_N_5_O_4_S_2_: C 57.79; H 4.07; N 13.48. Found: C 57.88; H 4.19; N 13.64.

*2-(4-(Hydroxy(phenyl)methyl)-1H-1,2,3-triazol-1-yl)-N-(6-(methylsulfonyl)benzo[d]-thiazol-2-yl)acetamide* (**5o**). Brown solid, m.p. 169–170 °C. IR (*υ*, cm^−1^): 1566 (C=C), 1602 (C=N), 1698 (C=O), 2963 (C-H al), 3069 (C-H ar), 3287–3360 (N-H, O-H). ^1^H-NMR: δ 3.19 (s, 3H, C**H**_3_), 5.59 (s, 2H, C**H**_2_), 5.79 (d, 1H, *J* = 4 Hz, CH), 6.23 (s, 1H, *J* = 8 Hz, O**H**), 7.25–7.41 (m, 5H, Ar-H), 7.99 (d, 1H, *J* = 8 Hz, Ar-**H**), 8.14 (d, 1H, *J* = 8 Hz, Ar-**H**), 8.17 (s, 1H, Ar-H), 8.68 (s, 1H, C**H**-1,2,3-triazole), 12.72 (s, 1H, N**H**). ^13^C-NMR: δ 41.3 (**C**H_3_), 50.2 (**C**H_2_), 69.2 (**C**-OH), 120.4, 121.8, 124.4, 127.1, 127.5, 128.6, 129.8, 141.2, 145.9, 147.0, 155.3, 163.7 (Ar-**C**, **C**=N, **C**=O) ppm. EI MS (*m*/*z*): 443.19 (M^+^). Anal. Calcd for C_19_H_17_N_5_O_4_S_2_: C 51.45; H 3.86; N 15.79. Found: C 51.31; H 3.70; N 15.68.

*N-(6-(Methylsulfonyl)benzo[d]thiazol-2-yl)-2-(4-(trimethylsilyl)-1H-1,2,3-triazol-1-yl)acetamide* (**5p**). Brown solid, m.p. 111–102 °C. IR (υ, cm^−1^): 1569 (C=C), 1600 (C=N), 1698 (C=O), 2910 (C-H al), 3024 (C-H ar), 3346 (N-H). ^1^H-NMR: δ 0.28 (s, 9H, 3 × C**H**_3_), 3.23 (s, 3H, C**H**_3_), 5.62 (s, 2H, C**H**_2_), 7.97 (d, 2H, *J* = 8 Hz, Ar-**H**), 8.21 (s, 1H, Ar-**H**), 8.67 (s, 1H, C**H**-1,2,3-triazole), 12.91 (s, 1H, N**H**). ^13^C-NMR: δ 0.3 (Si-**C**H_3_), 40.0 (**C**H_3_), 45.0 (**C**H_2_), 120.4, 121.7, 123.8, 128.5, 130.6, 135.4, 146.6, 156.7, 163.5 (Ar-**C**, **C**=N, **C**=O) ppm. EI MS (*m*/*z*): 409.13 (M^+^). Anal. Calcd for C_15_H_19_N_5_O_3_S_2_Si: C 43.99; H 4.68; N 17.10. Found: C 43.74; H 4.79; N 17.31.

*2-(4-(2-Hydroxyethyl)-1H-1,2,3-triazol-1-yl)-N-(6-(methylsulfonyl)benzo[d]thiazol-2-yl)acetamide* (**5q**). Brown solid, m.p. 248–249 °C. IR (υ, cm^-1^): 1588 (C=C), 1607 (C=N), 1701 (C=O), 2959 (C-H al), 3069 (C-H ar), 3268–3354 (N-H, O-H). ^1^H-NMR: δ 2.87 (t, 2H, *J* = 8 Hz, C-C**H**_2_), 3.27 (s, 3H, C**H**_3_), 3.61 (bt, 2H, OC**H**_2_), 4.91 (bs, 1H, O**H**), 5.49 (s, 2H, C**H**_2_), 7.88 (d, 1H, *J* = 8 Hz, Ar-**H**), 8.04 (d, 1H, *J* = 8 Hz, Ar-**H**), 8.19 (s, 1H, Ar-**H**), 8.71 (s, 1H, C**H**-1,2,3-triazole), 12.81 (s, 1H, N**H**). ^13^C-NMR: δ 29.6 (C-**C**H_2_), 40.7 (**C**H_3_), 51.9 (**C**H_2_), 60.8 (O**C**H_2_), 120.2, 121.9, 124.7, 128.0, 131.5, 145.6, 153.5, 158.8, 163.4 (Ar-**C**, **C**=N, **C**=O) ppm. EI MS (*m*/*z*): 380.93 (M^+^). Anal. Calcd for C_14_H_15_N_5_O_4_S_2_: C 44.08; H 3.96; N 18.36. Found: C 44.27; H 3.99; N 18.48.

*2-(4-(3-Hydroxypropyl)-1H-1,2,3-triazol-1-yl)-N-(6-(methylsulfonyl)benzo[d]thiazol-2-yl)acetamide* (**5r**). Brown solid, m.p. 267–268 °C. IR (υ, cm^−1^): 1568 (C=C), 1611 (C=N), 1721 (C=O), 2963 (C-H al), 3042 (C-H ar), 3299–3374 (N-H, O-H). ^1^H-NMR: δ 1.69–1.76 (m, 2H, OCH_2_C**H**_2_), 2.71 (t, 2H, *J* = 8 Hz, C-C**H**_2_), 3.32 (s, 3H, C**H**_3_), 3.52 (b, 2H, OC**H**_2_ overlapped with DMSO), 4.59 (s, 1H, O**H**), 5.51 (s, 2H, C**H**_2_), 7.84 (d, 1H, *J* = 8 Hz, Ar-**H**), 8.02 (d, 1H, *J* = 8 Hz, Ar-**H**), 8.15 (s, 1H, Ar-**H**), 8.70 (s, 1H, C**H**-1,2,3-triazole), 12.78 (bs, 1H, N**H**). ^13^C-NMR: δ 23.4 (OCH_2_**C**H_2_), 30.7 (C-**C**H_2_), 42.6 (**C**H_3_), 49.8 (**C**H_2_), 61.8 (O**C**H_2_), 119.8, 121.2, 124.4, 127.0, 131.2, 145.1, 148.7, 154.0, 157.2, 164.0 (Ar-**C**, **C**=N, **C**=O) ppm. EI MS (*m*/*z*): 395.20 (M^+^). Anal. Calcd for C_15_H_17_N_5_O_4_S_2_: C 45.56; H 4.33; N 17.71. Found: C 45.47; H 4.42; N 17.92.

### 3.5. Antimicrobial Activity

The antimicrobial inhibition potency of the newly synthesized benzothiazoles was estimated in terms of minimum inhibition concentration (MIC) by using the Broth Microdilution method [37,38]. Each compound was tested against clinical bacterial and fungal strains; (*Streptococcus pneumonia* RCMB 010010, *Bacillus subtilis* RCMB 010067, *Staphylococcus aureus* RCMB 010025, *Pseudomonas aeuroginosa* RCMB 010043, *Escherichia coli* RCMB 010052, *Klebsiella pneumonia* RCMB 010058, *Aspergillus fumigates* RCMB 02568 and *Candida albicans* RCMB 05036), and were obtained from the RCMB culture collection (Regional Center for Mycology and Biotechnology). The isolated clinical strains were subcultured on Mueller-Hinton Broth for bacteria and Sabouraud Liquid Broth for fungi. The stock solution of all compounds were prepared by dissolving 10 mg of the tested compound in dimethyl sulfoxide (DMSO, 1 mL). Progressive dilutions with distilled water gave the final concentrations of 1, 2, 4, 8, 16, 31.25, 62.5, 125, 250, and 500 mg·mL^−1^. All the inoculated tubes were incubated at 37 °C for 24 h.

## 4. Conclusions

This study reports on the synthesis of novel bioactive antibacterial and antifungal agents based on a 1,2,3-triazole-benzothiazole combined system under both conventional and ultrasound conditions. The synthesis approach required Cu(I)-catalyzed 1,3-dipolar cycloaddition coupling between the appropriate 2-azido-*N*-(benzo[*d*]thiazol-2-yl)acetamides with a variety of terminal alkynes, to afford regioselectively novel 1,2,3-triazoles tethering a benzothiazole moiety. Comparable to higher yields were obtained when the reactions were conducted under ultrasound irradiation with a significant reduction in the reaction times. The antimicrobial screening results revealed that the presence of the 1,2,3-triazole nucleus in the acetamido-benzothiazole scaffold resulted in increased antibacterial and antifungal activities.

## Figures and Tables

**Table 1:** Conventional *versus* ultrasound synthesis of compounds **2a**–**c** and **3a**–**c**.

Compound No.	R	Classical Method CM		Ultrasound Method US	
		Time (h)	Yield (%)	Time (h)	Yield (%)
**2a**	H	2	88	1	92
**2b**	CH_3_	2	86	1	91
**2c**	CH_3_SO_2_	3	83	1	89
**3a**	H	24	92	2	96
**3b**	CH_3_	24	91	2	96
**3c**	CH_3_SO_2_	24	88	3	94

**Table 2:** Conventional *versus* ultrasound synthesis of 1,2,3-triazoles **5a**–**r**.

Compound No	Structure	Classical Method CM		Ultrasound Method US	
		Time (h)	Yield (%)	Time (h)	Yield (%)
**5a**	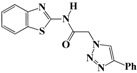	6	90	3	96
**5b**	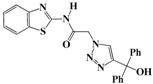	8	88	4	94
**5c**	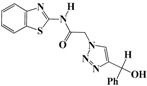	7	89	4	96
**5d**	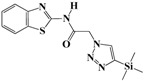	9	85	5	90
**5e**	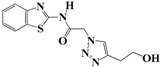	8	87	3	93
**5f**	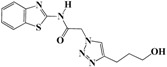	8	87	3	92
**5g**	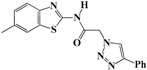	6	89	3	96
**5h**	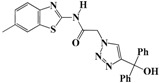	8	88	4	93
**5i**	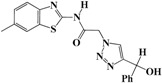	7	88	4	95
**5j**	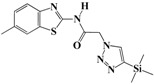	10	84	5	89
**5k**	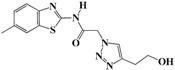	8	87	3	92
**5l**	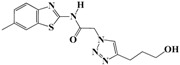	8	87	4	92
**5m**	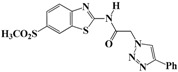	8	81	4	92
**5n**	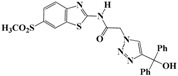	9	84	5	89
**5o**	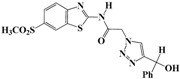	8	85	5	91
**5p**	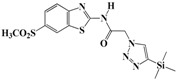	10	81	6	87
**5q**	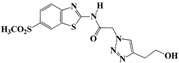	9	83	4	89
**5r**	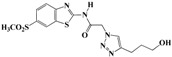	9	82	5	88

**Table 3:** Antimicrobial activity expressed as Minimum Inhibition Concentration (MIC, μg/mL).

Compound No	Gram-Positive Organisms			Gram-Negative Organisms			Fungi	
	*Sp*	*Bs*	*Sa*	*Pa*	*Ec*	*Kp*	*Af*	*Ca*
**2a**	31.25	31.25	16	31.25	16	31.25	125	62.5
**2b**	31.25	31.25	16	16	16	31.25	125	125
**2c**	16	31.25	16	16	16	16	125	62.5
**3a**	31.25	16	16	16	16	31.25	62.5	31.25
**3b**	31.25	16	16	16	16	31.25	31.25	31.25
**3c**	16	16	16	16	16	16	31.25	31.25
**5a**	16	16	16	16	16	16	31.25	31.25
**5b**	16	16	16	16	16	16	31.25	31.25
**5c**	16	8	8	16	8	16	16	16
**5d**	16	16	8	8	8	16	31.25	31.25
**5e**	8	8	8	8	8	16	16	16
**5f**	8	8	8	8	8	16	16	16
**5g**	16	16	16	16	16	16	31.25	31.25
**5h**	16	16	16	16	16	16	31.25	31.25
**5i**	8	8	8	16	8	8	16	16
**5g**	16	8	8	8	8	8	31.25	31.25
**5k**	8	8	8	8	8	8	16	16
**5l**	8	8	8	8	8	8	16	16
**5m**	8	8	16	8	8	16	16	16
**5n**	8	16	8	8	8	16	16	16
**5o**	8	8	4	4	8	8	8	8
**5p**	8	8	8	16	8	8	16	8
**5q**	4	4	8	4	4	8	4	4
**5r**	4	4	8	4	4	8	4	4
Ciprofloxacin	≤4	≤1	≤4	≤4	≤1	≤1	-	-
Fluconazole	-	-	-	-	-	-	≤1	≤1

*Sp*: *Streptococcus pneumonia*; *Bs*: *Bacillus subtilis*; *Sa*: *Staphylococcus aureus*; *Pa*: *Pseudomonas aeuroginosa*; *Ec*: *Escherichia coli*; *Kp*: *Klebsiella pneumonia*; *Af*: *Aspergillus fumigates*; *Ca*: *Candida albicans*.
